# 100 days of Adolescence: Elucidating Externalizing Behaviors Through the Daily Assessment of Inhibitory Control

**DOI:** 10.1007/s10802-023-01071-y

**Published:** 2023-07-05

**Authors:** Natasha Chaku, Ran Yan, Dominic P. Kelly, Zhuoran Zhang, Nestor Lopez-Duran, Alexander S. Weigard, Adriene M. Beltz

**Affiliations:** Department of Psychology, University of Michigan, Ann Arbor, MI USA

**Keywords:** Adolescence, Group Iterative Multiple Model Estimation, Impulsivity, Intensive longitudinal design, Stroop Color Word Task, Substance use

## Abstract

Inhibitory control is a transdiagnostic risk factor for externalizing behaviors, particularly during adolescence. Despite advances in understanding links between inhibitory control and externalizing behaviors across youth *on average*, significant questions remain about how these links play out in the day-to-day lives of individual adolescents. The goals of the current study were to: (1) validate a novel 100-occasion measure of inhibitory control; (2) assess links between day-to-day fluctuations in inhibitory control and individual differences in externalizing behaviors; and (3) illustrate the potential of intensive longitudinal studies for person-specific analyses of adolescent externalizing behaviors. Participants were 106 youth (57.5% female, *M*_*age* _= 13.34 years; *SD*_*age *_= 1.92) who completed a virtual baseline session followed by 100 daily surveys, including an adapted Stroop Color Word task designed to assess inhibitory control. Results suggested that the novel task was generally reliable and valid, and that inhibitory control fluctuated across days in ways that were meaningfully associated with individual differences in baseline impulsive behaviors. Results of illustrative personalized analyses suggested that inhibitory control had more influence in the daily networks of adolescents who used substances during the 100 days than in a matched set of adolescents who did not. This work marks a path forward in intensive longitudinal research by validating a novel inhibitory control measure, revealing that daily fluctuations in inhibitory control may be a unique construct broadly relevant to adolescent externalizing problems, and at the same time, highlighting that links between daily inhibitory control and impulsive behaviors are adolescent-specific.

**Supplementary Information:**

The online version contains supplementary material available at 10.1007/s10802-023-01071-y.

Behaviors on the externalizing spectrum, such as impulsivity, substance use, and conduct problems, increase during adolescence. Although this increase is generally considered normative, individual differences in externalizing behaviors can result in social difficulties, school failure, and persistent deviance (Odgers et al., 2008). Inhibitory control (IC; the ability to suppress prepotent responses) may play a key role in regulating externalizing behaviors (Young et al., 2009). This field of research, however, has overwhelmingly relied on cross-sectional or a few longitudinal assessments, and typically represents IC as a trait or locally stable construct. Yet, growing evidence suggests that cognition fluctuates at granular timescales and is meaningfully linked to personal characteristics and wellbeing (Brose et al., 2014; Sliwinski et al., 2006). Thus, it is likely that IC is associated with externalizing behaviors in unique ways for individual adolescents, but that the research methods employed to-date could not empirically detect this.

Intensive longitudinal designs can be used to understand cognitive fluctuations and their psychological significance. These methods involve many repeated assessments of the same variables and participants over relatively short periods of time: from moment-to-moment (e.g., neural activity; Demidenko et al., 2022), hour-to-hour (e.g., affect; Brose et al., 2014), or day-to-day (e.g., personality; Kelly et al., 2020), revealing how behavior, including cognition, unfolds within an individual. Intensive longitudinal designs can also support a range of inferences, including generalizable nomothetic inferences made from examinations of interindividual (i.e., between-person) variation, idiographic inferences about unique individuals made from examinations of intraindividual (i.e., within-person) variation, or a combination of the two (Molenaar, 2004).

Unfortunately, there is a lack of cognitive measures that are well-suited for intensive longitudinal assessment, potentially because highly controlled, lab-based measurement is at odds with mobile, ecologically-valid assessments (Roche et al., 2016). Thus, the psychometric validation of intensive longitudinal cognitive assessments is a challenging, nascent area of research (but see Kelly & Beltz 2021; Sliwinski et al., 2018). The goals of the current study were to: (1) develop and validate a novel 100-occasion measure of IC; (2) assess how daily IC fluctuations were related to baseline impulsive behaviors; and (3) explicate the potential of idiographic analyses for revealing adolescent-specific links among daily IC and externalizing behavior in social contexts.

## IC is a Risk Factor for Adolescent Externalizing Behaviors

IC is one of the most salient cognitive factors associated with externalizing behaviors (Young et al., 2009). Although most work has focused on children (see Schoemaker et al., 2013), IC plays an important role in adolescence, too. Deficits in IC reliably mark adolescent populations with clinically relevant problem behaviors. For instance, low IC predicts early onset of alcohol use-related problems (López-Caneda et al., 2014), illicit drug use (Nigg et al., 2006), and high likelihood of antisocial or oppositional defiant disorder diagnoses (Bonham et al., 2021). Even in non-clinical samples, low IC is consistently linked with increased externalizing behaviors in both cross-sectional and longitudinal research (Kim-Spoon et al., 2019).

IC may be particularly important for understanding adolescent behaviors because it continues to mature into early adulthood (Luna et al., 2015) and occurs in the context of early adolescent increases in neural reactivity to socioemotional stimuli (Foulkes & Blakemore, 2018), which may contribute to normative rises in externalizing behaviors (Lydon-Staley & Bassett, 2018). Most studies on this topic to-date are cross-sectional and limited to nomothetic inferences even though they assume cognitive control and socioemotional reactivity change *within individuals over time* (see Beltz 2018). Thus, there is theoretical acknowledgement of the importance of *intra*individual variation for adolescent externalizing behaviors, but there is little empirical evidence for it, as the extant literature primarily concerns *inter*individual variation (Chaku & Beltz, 2022). Intensive longitudinal research is needed to fill this critical knowledge gap.

## From Nomothetic to Idiographic Assessments of IC

IC is often conceptualized as a relatively stable trait that can be assessed once and generalized across time. Converging evidence increasingly shows, however, that short-term fluctuations in cognition have significance for psychological wellbeing (Castellanos et al., 2005; Hultsch et al., 2011). Numerous factors, such as interpersonal experiences, contextual demands, and emotional processes, can influence or be influenced by cognition at any given moment. For example, increased working memory fluctuations (measured across four weeks with four assessments a day) have been associated with poor sleep quality in early adolescents (Galeano-Keiner et al., 2022). Similarly, better working memory (measured daily over two weeks) has been associated with less negative mood and greater school belonging in late adolescence (Wang et al., 2021).

Unfortunately, work in this area is challenged by a lack of sufficient measurement tools. Current cognitive assessments (e.g., Stroop Color Word, Stop-Signal, and Go/No Go tasks) have primarily been used to capture normative IC-linked processes, but they have limitations (see Snyder et al., 2015). Specifically, recent work suggests that the subtraction methods often used to analyze recorded task data (e.g., comparing reaction times on incongruent versus congruent trials) are unreliable and do not index individual differences well (Salthouse & Hedden, 2002; Weigard et al., 2021), which limits their suitability for studies of intraindividual variation.

Nonetheless, understanding short-term cognitive fluctuations is essential because patterns of intraindividual variability may reliably differ across individuals, potentially reflecting an enduring personal characteristic (Nesselroade, 1991). Although the extent to which fluctuations may reflect vulnerability versus resilience, and in turn adjustment, likely depends on the specific cognitive construct, context, or sample, the general importance of cognitive variation is being increasingly realized. For instance, greater response variability on a lab-based cognitive task was associated with lower perceptions of real-world risks (e.g., substance use) in 14-to-16-year-olds (Goldenberg et al., 2017). Yet, most studies only capture trial-to-trial fluctuations during a single, highly controlled task, so little is known about how longer-term fluctuations in IC are associated with externalizing behaviors in everyday life. This is unfortunate because *daily* fluctuations are likely related to adolescents’ unique but recurring biopsychosocial experiences.

Beyond understanding fluctuations in cognition, daily data (if abundant) can also be used to make person-specific inferences. Adolescence is characterized by increased, variable opportunities for learning, adaptation, and exploration (Knoll et al., 2016). These experiences do not just differ in degree, but also in nature and biopsychosocial context across individual adolescents (Chaku & Beltz, 2022). To capture this heterogeneity, averages (and even average fluctuations) must be eschewed in favor of person-specific models (Molenaar, 2004). For instance, group iterative multiple model estimation (GIMME; Gates & Molenaar 2012) is a person-specific analytic technique that maps variation among pre-selected variables within a person over many relatively short intervals. Applied to daily adolescent behavior, GIMME considers each adolescent as if they are a sample of *N*=1, allowing for unique inferences about the timing of relations among variables (i.e., same-day or next-day) that potentially reflect adolescents’ real-world contexts. GIMME is unique among person-specific analytic approaches because it also affords some generalizable inferences. It does so without averaging by prioritizing relations that are common across a sample. Thus, when combined with daily data, GIMME and other person-specific techniques can be used to make potentially important insights into *when*, *for whom*, and *in what situations* links between IC and externalizing behaviors matter.

## Intensive Longitudinal Measures of IC

The feasibility of intensive longitudinal studies is increasing due to wide accessibility of internet-capable devices and sophisticated analytic techniques for dependent data (Lydon-Staley & Bassett, 2018). Still, there is a paucity of *cognitive* assessments available for inclusion in these studies. Beyond their traditionally restricted use in laboratories, this may be because cognitive measures cannot be intensively repeated due to trial counts, length, or practice effects, or because they require timing mechanisms that are difficult to apply in situ (Ladouce et al., 2016).

Nonetheless, a few intensive cognitive assessments have been validated. For example, Sliwinski and colleagues (2018) validated working memory (i.e., dot memory and n-Back) and perceptual speed (i.e., symbol search) tasks across 14-day ecological momentary assessment (EMA) bursts. Although links to other behaviors were not examined, the working memory task exhibited lower intraindividual reliability than the perceptual speed task. Recently, Kelly and Beltz (2021) validated novel, openly available 75-occasion measures of delayed verbal recall (https://osf.io/vhr7u/) and three-dimensional (3D) mental rotations (https://osf.io/m6ae8/). This involved creating 75 unique sets of stimuli and establishing procedures for parallel forms reliability across sets. Overall, the 3D mental rotations measure had better psychometrics than the delayed verbal recall measure, however, there are significant challenges in distinguishing between unreliability and meaningful fluctuation in intensive longitudinal measurement (Keijsers et al., 2022). Thus, the authors posed that verbal recall may be more influenced by daily experiences compared to mental rotations but did not empirically examine this possibility. This small literature suggests that intensive longitudinal measurement of cognition is feasible, but it has not yet been widely employed to study adolescent IC in everyday contexts.

## The Current Study

IC likely varies from day-to-day in ways that have significance for externalizing behaviors in unique adolescents, but measures are not yet available to examine this. Thus, the overarching study goal was to reveal adolescents’ daily associations between IC and externalizing behaviors through three aims. First, a 100-occasion measure of IC was developed and psychometrically validated. Second, daily IC (both 100-day averages and fluctuations) were related to baseline impulsive behaviors; low averages and high fluctuations were expected to predict impulsivity. Third, the potential of person-specific analyses for revealing links between IC and externalizing behaviors was illustrated by comparing the daily networks of adolescents who used substances to the networks of a matched subsample who did not.

## Method

Data come from a 100-day intensive longitudinal study of adolescent daily experiences. The study protocol was approved by University of Michigan Institutional Review Board for Health Sciences and Behavioral Sciences (IRB-HSBS). No data have been previously reported.

### Participants

The final sample included 106 adolescents (57.5% girls, 38.7% boys, 3.8% other gender identity^1^). Adolescents were between 9 and 18 years old (*M* = 13.34, *SD* = 1.92), and 75% were at least 12 years old. Most were White (75.5%) and not Latin(o/a) (91.5%), with others identifying as more than one race (17.9%), Black/African American (5.7%), or American Indian/Alaskan Native (0.9%). Families were recruited through social media, virtual flyers, and university-affiliated databases. Families were defined as one parent or legal guardian and two children (of any degree of genetic relatedness) between 8 and 21 years old, with at least one child between age 8 and 17. After enrollment, some adolescents did not complete the study or meet inclusion criteria for this paper (see below). Thus, the final sample reported here consists of 63 families with 43 sibling pairs and 20 singletons. The average daily response rate of included adolescents was 94%.

The full study sample consisted of 174 adolescents (54.9% girls, 42.9% boys, 2.2% other gender identity) aged 8 to 20 years old (*M* = 13.37, *SD* = 2.19). Of these adolescents, 6.3% withdrew from the study, 13.3% were dropped from the study because they failed to complete at least 50% of the daily diaries after the first 30 days, and 19.5% completed the study but were excluded from this paper because their response rate was less than 80%. This follows procedures and simulation results from past research on data fidelity and missingness (Rankin & Marsh, 1985; Wright et al., 2019). Included participants did not differ from excluded participants demographically (i.e., gender, age, or race/ethnicity, all *p*s ≥ 0.05), however, they did report fewer impulsive behaviors as well as greater working memory and attentional control (all *p*s ≤ 0.01); they did not report differences in perceptual sensitivity (*p* > .05).

### Procedure

The study was conducted virtually between March 2021 and August 2022. Families first completed a baseline session in which parents provided electronically signed informed consent for themselves and for their children under 18 years old, who also provided informed assent. Adolescents aged 18 years or older provided informed consent. Parents and adolescents then independently completed 90-minute online surveys (using any Internet-capable device) via Qualtrics. Surveys contained questions about their identities, feelings, and behaviors as well as cognitive tests. Then, every night for the next 100 days, adolescents completed a 20-minute online survey. Around 5:00PM, unique survey links were sent to the parent’s email address^2^, who distributed them to their children. Adolescents were asked to take the survey at 8:00PM or after that day’s activities; survey links expired the next day at noon. Among other measures, the daily surveys included questions about externalizing behaviors and social experiences as well as the novel IC task. Each family member received $15 for completing the baseline survey. Adolescents received $1 for each completed daily survey, which doubled to $2 if they completed at least 80% of the surveys; they received a $35 bonus if they completed at least 90% of the daily surveys.

### Measures

The focus of this paper is on the novel intensive longitudinal measure of IC. Baseline measures of cognition (i.e., task-based working memory and self-reported attentional control) and age were used to assess convergent validity; measures of perceptual sensitivity and gender were used to assess discriminant validity. To examine how IC’s daily average and fluctuations were linked to externalizing behaviors, baseline impulsive behaviors were used. Daily impulsive behaviors and social experiences were also used in illustrative person-specific analyses.

#### Baseline Measures

**Task-Based Working Memory.** The Symmetry Span task (Foster et al., 2015) was used to assess working memory. Adolescents were shown a series of highlighted red squares in a 4 × 4 black and white matrix. Interspersed was a symmetry task in which adolescents had to judge whether a pattern composed of black and white squares was symmetrical along its vertical axis. Set lengths ranged from 2 to 5 symmetry-matrix combinations (12 trials total). At the end of each set, adolescents were asked to recall the location of each red square in the correct order. Adolescents received two practice trials and were required to maintain at least 85% accuracy on the symmetry trials. The absolute span score (i.e., sum of all perfectly recalled sets) was used in the subsequent analyses (Shipstead et al., 2012).

**Self-Reported Attentional Control.** Attentional control was assessed using a subscale from the Early Adolescent Temperament Questionnaire-Revised (EATQ-R; Ellis & Rothbart 1999). It contained six items on capacity to focus and shift attention (e.g., “It is easy for me to really concentrate on homework problems”; α = 0.75). Items were rated on a 5-point scale (1 = *Almost always untrue* to 5 = *Almost always true*) with higher scores reflecting greater control.

**Self-Reported Perceptual Sensitivity.** Perceptual sensitivity was assessed using another subscale from the EATQ-R (Ellis & Rothbart, 1999). It contained four items on the detection or awareness of slight, low-intensity stimulation in the environment (e.g., “I am very aware of noises.”; α = 0.81). Higher scores reflect greater sensitivity.

**Self-Reported Impulsive Behaviors.** Impulsive behaviors were assessed with the 40-item UPPS-P Impulsive Behavior scale (Lynam et al., 2006). It has five subscales, measuring a lack of premeditation (e.g., “I am one of those people who blurt out things without thinking”; α = 0.76), lack of perseverance (e.g., “I tend to give up easily”; α = 0.76), sensation seeking (e.g., “I quite enjoy taking risks”; α = 0.87), positive urgency (e.g., “I tend to act without thinking when I am really excited”; α = 0.93), and negative urgency (e.g., “When I feel bad, I will often do things I later regret in order to make myself feel better now”; α = 0.89). Items were rated on a 4-point scale (1 = *Not at all like me* to 4 *= Very much like me*) with higher scores reflecting greater impulsivity.

#### Daily Measures

**IC.** The Stroop Color Word Test (Golden, 1975) was adapted for the 100 daily assessments. In the classic measure, participants received 3 pages with 100 color words each (“red”, “green”, “blue”). On the first page, all color words were printed in black ink. On the second page, all color words were printed in congruent ink (e.g., the word “red” printed in red ink), and on the third page, all color words were printed in incongruent ink (e.g., “red” printed in green ink). Answer choices included each color word printed in black ink. Participants were given 45 seconds per page to circle as many answers corresponding to the ink color as possible. It was expected that more items would be correctly circled on congruent than incongruent pages; the number of correctly answered incongruent items was the IC score.

Since 1975, subtraction methods (e.g., comparing reaction time on incongruent and congruent trials) have gained popularity, but they have significant limitations for accurately detecting individual differences (Draheim et al., 2019; Weigard et al., 2021), and thus, intraindividual variation. Therefore, in this study, adolescents were presented with a randomized set of 100 color words (“red”, “green”, blue”, or “yellow”) in incongruent colors only (e.g., “red” in green font). Consistent with other work (Heitz & Engle, 2007), there were no neutral or congruent conditions, and all combinations of words and font colors were presented the same number of times per day. As in the classic measure, adolescents indicated the font color of as many words as they could in 45 seconds by selecting the correct color word presented in black ink (see supplemental materials for task images)^3^. The number of correct responses indexed each day’s IC score; this is consistent with the original measure and has been suggested as a viable alternative to reaction time-based indices (Khng & Lee, 2014). On the first day, adolescents also completed five practice trials with feedback. The task is openly available (https://osf.io/9yabr/).

To ensure data fidelity, some trials were excluded for some adolescents. Days adolescents completed one or fewer trials correctly were excluded (<0.5%), as they likely reflected low effort or technical issues. Also, days adolescents completed nearly all 100 trials were winsorized to three standard deviations above that day’s average (<0.5%), as they likely reflected technical issues (e.g., screen freezing) and internal testing suggested it would be difficult to complete more than 75 trials.

**Daily Impulsive and Social Experiences.** Daily positive urgency and daily negative urgency (i.e., tendencies to act impulsively when experiencing positive and negative emotions, respectively) were assessed via the Short UPPS-P (Cyders et al., 2014); each scale contained four items adapted to reflect adolescents’ impulsive behaviors that day (e.g., positive urgency: “*Today*, I tended to act without thinking when I was really excited”) and were rated on a 4-point scale (1 = *Not at all like me* to 4 = *Very much like me*). Similar measures have been adapted and used in intensive longitudinal studies (Sperry et al., 2016; Tomko et al., 2014). Reliabilities were good, according to multilevel confirmatory factor analysis (Schuurman & Hamaker, 2019). For daily positive urgency, between-person ω = 0.80 and within-person ω = 0.79. For daily negative urgency, between-person ω = 0.81 and within-person ω = 0.88.

Daily social experiences were assessed using a modified activity questionnaire (McHale et al., 1999). Adolescents completed one item indicating how much time (in minutes) they spent visiting or hanging out that day on a sliding scale from 0 to 100. Responses were binned: 0 = *Did not visit or hang out today*; 2 (1–49 min) = *Visited or hung out a little*; 3 (50–99 min) = *Visited or hung out a moderate amount*; 4 (100 + minutes) = *Visited or hung out a lot*. Further details for the daily measures are provided in the supplemental materials.

### Analytic Plan

Three sets of analyses were conducted. First, the reliability and validity of the novel IC measure were assessed. Second, average daily IC scores and fluctuations in those scores were associated with baseline impulsive behaviors. Finally, personalized network analyses (using GIMME; Gates & Molenaar 2012) were conducted for a subset of individuals to illustrate the utility of intensive longitudinal data for person-specific inferences. Analyses were conducted in SPSS (version 26) and R (v4.1.2; R Core Team, 2022).

**Reliability and Validity of Daily IC.** Parallel forms reliability was assessed by comparing the interindividual IC means and standard deviations (*SD*s) across all 100 days. Daily means and *SD*s across participants for each day were expected to be approximately equal and normally distributed, suggesting that random differences in stimuli order did not systematically impact IC assessment. Intraclass correlation coefficients (ICCs) were also calculated, with values greater than 0.50 indicating moderate reliability, 0.75 good reliability, and 0.90 excellent reliability (Bartko, 1966). Moderate to good reliability across days was expected (see Kelly & Beltz 2021).

Convergent validity was assessed by correlating each day’s IC score with standard baseline measures of cognition (i.e., working memory and attentional control) across participants. Low to moderate correlations were expected because they assessed similar, but distinct domains of cognition, and in the case of attentional control, in a different modality (Toplak et al., 2013). Convergent validity was also assessed by correlating each day’s IC score with age. Older youth were expected to have higher IC. Discriminant validity was assessed by correlating each day’s IC score with baseline perceptual sensitivity and via gender differences. No relations were expected given weak, null, and inconsistent findings in the extant literature (Weafer, 2020). All reliability and validity analyses used listwise deletion for daily missing data, but there were at least 92 participants (87%) included in each day’s analysis. Effect sizes for independent analyses were evaluated using *r* and Cohen’s *d*, with small effect sizes corresponding to *r* = 0.1 and *d* = 0.2, medium to *r* = 0.3 and *d* = 0.5, and large to *r* = 0.5 and *d* = 0.8 (Cohen, 1988). Nested analyses (accounting for family dependencies) showed the same pattern of results as the independent analyses described in the main text; they are available in the supplemental materials.

**Fluctuations in Daily IC and Links to Impulsive Behaviors.** Each adolescent’s average or mean IC (i*M*) across the 100 days was calculated. Fluctuations were calculated using intraindividual standard deviations (i*SD*); smaller i*SD*s reflect fewer and/or smaller deviations from an adolescent’s own average, and larger i*SD*s reflect more and/or larger deviations from that average. For instance, an adolescent with i*M* = 34 and i*SD* = 2.7 demonstrates relatively consistent performance across days compared to an adolescent with i*M* = 34 and i*SD* = 7.5 who has the same level of performance but with larger variability, sometimes scoring well below or above their average. A one-sample *t*-test was used to determine whether the sample showed fluctuations (i.e., i*SD*s significantly different from zero). Gender and age effects were also explored. Multilevel models (nesting individuals within families) were then used to assess associations between baseline impulsive behaviors (i.e., lack of premeditation, lack of perseverance, sensation seeking, positive urgency, and negative urgency) and daily IC (i*M*, i*SD*). Each outcome was assessed in a separate model and all models included age and gender (0 = boys; 1 = girls)^4^.

**Illustrative Adolescent-Specific Network Analyses**. Finally, illustrative adolescent-specific network analyses were conducted via GIMME to highlight the utility of intensive longitudinal methods for future work on externalizing behaviors. Specifically, 12 adolescents who reported any substance use (e.g., tobacco, marijuana, or alcohol) during the 100-day study were matched (see supplemental materials) with 12 adolescents who reported no substance use. This extreme groups comparison is ideal for highlighting heterogeneity among adolescents who use substances (and demographically similar youth who do not). Their daily IC, positive urgency, negative urgency, and social time were linearly detrended by day (as many time series approaches assume stationarity; Beltz & Gates 2017), and then submitted to confirmatory subgrouping-GIMME (CS-GIMME; Henry et al., 2019).

**Fig. 1:**
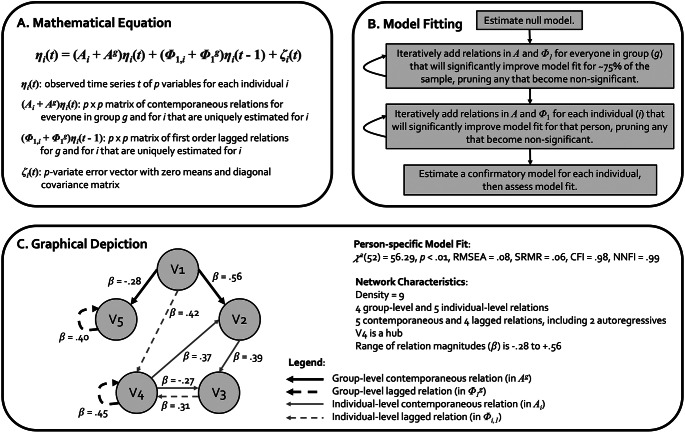
Mathematical equation, model fitting procedure, and graphical depiction of standard GIMME, which contains group- and individual-level relations; the extension of GIMME used in this paper also included confirmatory subgroup-level relations (see Henry et al., 2019). Adapted with permission from Chaku, N., & Beltz, A. M. (2022). Using temporal network methods to reveal the idiographic nature of development. *New Methods and Approaches for Studying Child Development*, 159

CS-GIMME is a variant of GIMME which uses unified structural equation models (or uSEMs) in combination with a grouping algorithm to derive sparse, person-specific networks of directed relations among intensively measured variables (Gates & Molenaar, 2012). It is unique among network approaches because it estimates contemporaneous (i.e., same day) and first order lagged (i.e., next-day) relations, providing some temporal indexing for relations, and because it provides both nomothetic and idiographic inferences via relations that can apply to the whole sample or to a single adolescent. As described in Fig. 1, GIMME derives person-specific networks through a multistep, data-driven process. The analysis begins with a null model. Then, a directed relation is added between two variables if it would significantly improve model fit for at least 75% of the sample (as determined by Lagrange Multiplier tests; Sörbom 1989); these group-level relations are iteratively added until none meets the 75% criterion. In this analysis, autoregressive relations (i.e., variables predicting themselves from one day to the next) are specified at the group-level to facilitate model fitting (a common procedure; Lane et al., 2019). Individual-level relations are then added if an individual’s model does not fit well with only group-level relations. After each level is fit, models are pruned of relations that no longer meet criteria, and final models are evaluated using standard fit indices. In this study, CS-GIMME was used to permit *a priori* comparisons between adolescents who did and did not use substances over the 100 days. In CS-GIMME (Henry et al., 2019), subgroup-level relations are iteratively estimated after group-level relations and before individual-level relations (with a 51% criterion). This means that relations common among the majority of youth who used substances have the opportunity to be estimated separately from youth who did not use substances. Thus, each resulting network reflects a personalized set of relations with unique weights (some of which apply to everyone, some of which apply only to an adolescent’s subgroup, and some of which are unique to an adolescent). GIMME uses full information maximum likelihood, and only includes individuals with 80% or more of the daily dairies. GIMME, and its extensions, have been widely used and are well-supported by largescale simulations (see Gates & Molenaar 2012; Henry et al., 2019; Lane et al., 2019).

After GIMME, network node centrality was calculated and compared across subgroups. Node centrality is the number of relations for each variable divided by the total number of network relations. It reflects the relative influence of each behavior in an adolescent’s person-specific model (Beltz & Gates, 2017). Independent sample *t*-tests were conducted to determine whether node centrality differed across adolescents who used substances and those who did not.

## Results

### Reliability and Validity of Daily IC

**Fig. 2:**
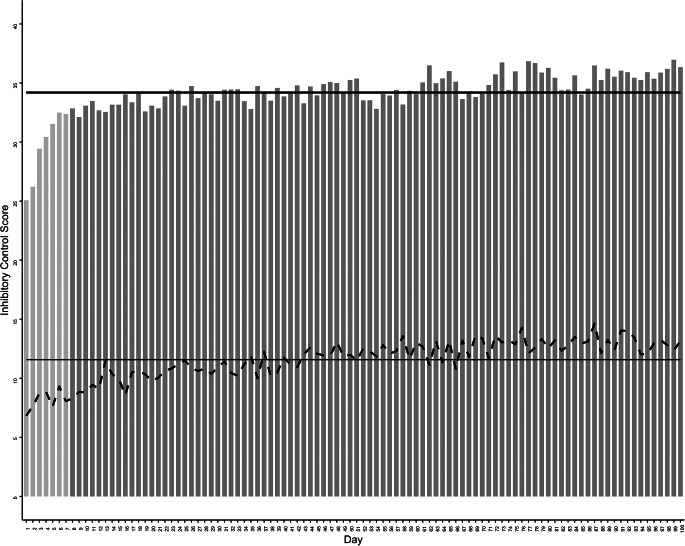
Daily means and standard deviations of the novel inhibitory control (IC) measure, across adolescent participants (*N* = 106). Each bar represents the average score for each of the 100 study days. The thick black line represents the average score across all participants and all study days. The dashed black line represents each day’s standard deviation, reflecting variability between participants. The thin black line represents the average standard deviation across participants and all study days. Means for the first seven days are depicted with light gray bars to emphasize performance differences early in the study

The interindividual means and *SD*s of IC across 100 days are shown in Fig. 2. Each bar represents *that day’s* IC averaged across all adolescents. The thick black line represents the overall IC score (*M* = 34.19), aggregated across adolescents and days, and the thin dashed line represents daily variation across adolescents’ scores (overall *SD* = 1.82).

Overall skewness was 2.23 and kurtosis was 8.84, suggesting that the distribution was negatively skewed and heavy-tailed. This is easy to visualize in Fig. 2; IC notably increases over the first week (indicated by light gray bars). Excluding this week, the mean for the remaining 93 days was similar, and *SD* expectedly decreased (*M* = 34.53; *SD* = 1.14); the skewness and kurtosis also decreased to close to zero (skewness: 0.18, kurtosis: − 0.70), approximating a normal distribution. The ICC was 0.53 (95% CI = [0.47, 0.61]), suggesting relatively good reliability across days, and it was 0.56 (95% CI = [0.50, 0.63]) across the last 93 days.

**Fig. 3:**
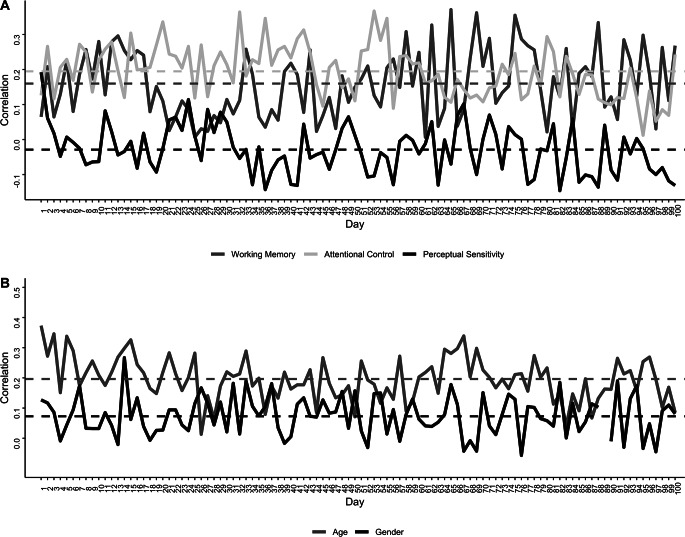
Average daily correlations between the novel inhibitory control (IC) measure and standard baseline measures: working memory (dark grey), attentional control (light grey), and perceptual sensitivity (black)

Convergent and divergent validity were assessed via correlations of daily IC with standard baseline measures. These correlations are shown in Fig. 3. Regarding convergent validity, there were small-to-moderate, positive correlations. IC’s average correlation with working memory (Fig. 3, dark gray) was *r* = .16 (*SD* = 0.10; range: 0.03 - 0.37), and 37% of the correlations were significant at *p* < .05. IC’s average correlation with attentional control (Fig. 3, light gray) was *r* = .20 (*SD* = 0.07; range: 0.01 - 0.37), and 54% were significant. IC’s average correlation with age was *r* = .20 (*SD* = 0.07; range: 0.01 - 0.38), and 52% were significant; thus, older adolescents had higher IC. Regarding divergent validity, perceptual sensitivity (Fig. 3, black) was not significantly correlated with any day’s IC performance (average *r *= -.03, all *p*s > 0.05). On average, girls (*M* = 35.03; *SD* = 11.72) had better daily IC than boys (*M* = 33.43; *SD* = 11.30), but this difference was not significant (*p* > .05), and girls only significantly outperformed boys on a single day, which likely reflects Type I error. The average effect size (Cohen’s *d*) across all 100 days was small (*d* = 0.15; *SD* = 0.11; range: 0.002 - 0.56). Each daily correlation and multilevel models of the same relations adjusting for family dependencies are provided in supplemental materials.

## Fluctuations in Daily IC and Links to Impulsive Behaviors

**Fig. 4:**
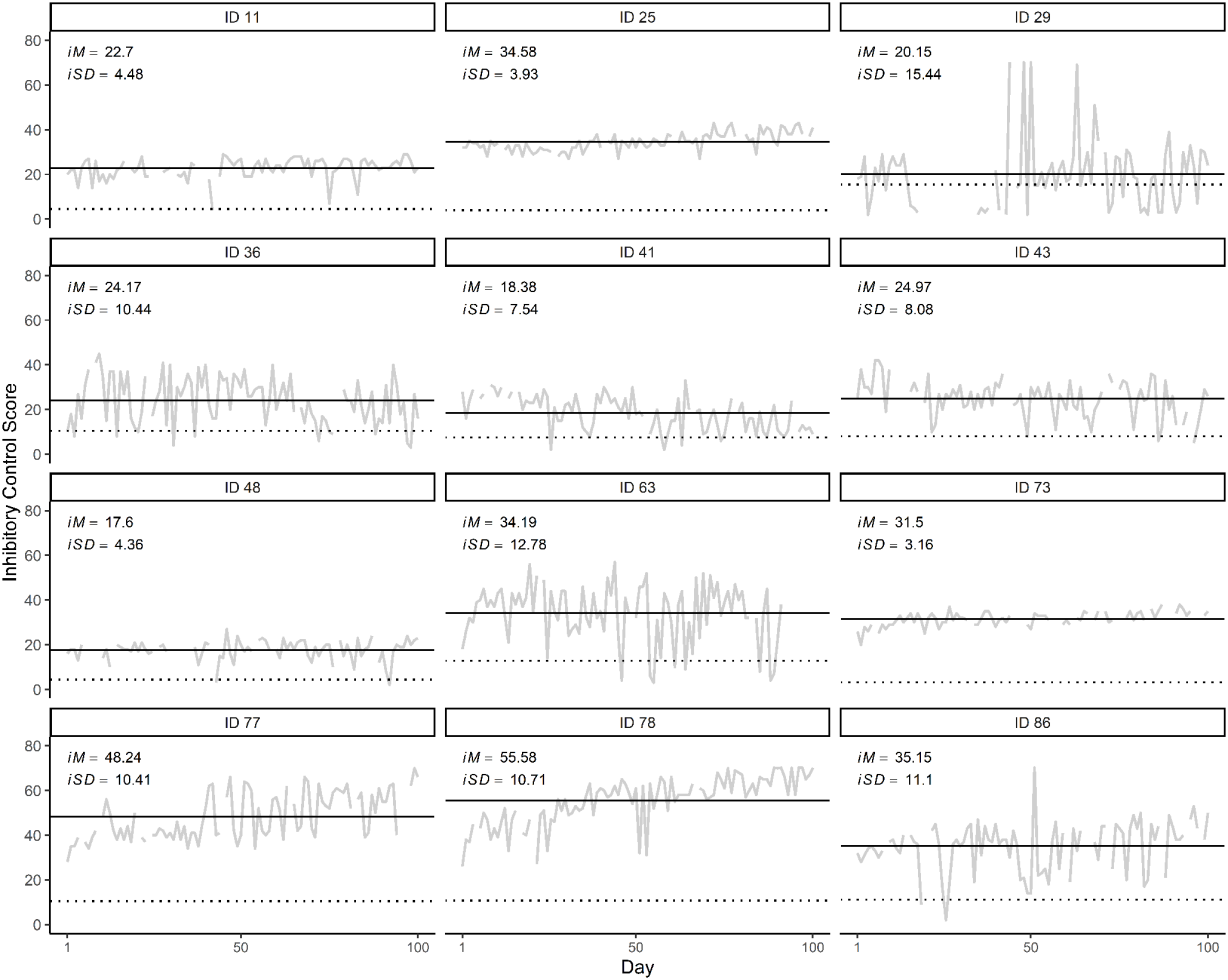
Plots of daily inhibitory control (IC) for 12 illustrative participants. Each plot shows the intensive longitudinal study data for one adolescent, with the IC score on the y-axis and study day on the x-axis. Grey lines show raw scores. Dashed black lines show i*M*s (i.e., each adolescent’s average IC score across all days), and dotted black lines show i*SD*s (i.e., each adolescent’s standard deviation across all days)

Daily IC scores for 12 illustrative adolescents are shown in Fig. 4 along with their i*M*s and i*SD*s. Adolescents demonstrated remarkably different trajectories. For example, ID 36 exhibited large day-to-day variation (i*SD* = 10.44), whereas the ID 73 exhibited far less variability (i*SD* = 3.16). Further, some adolescents were characterized by a relatively flat trajectory (e.g., ID 11) while others were better characterized by positive (e.g., ID 77), negative (e.g., ID 41), or non-linear (e.g., ID 29) trajectories. Across adolescents, the i*M* ranged from 12.79 to 55.58, and the i*SD* ranged from 3.16 to 15.44. A one-sample *t*-test indicated that the i*SDs* were significantly different from zero, *t*(105) = 31.53, *p* < .001, providing statistical evidence for IC fluctuations. The i*M* and i*SD* were not significantly correlated (*r *= -.01, *p* = .91). Although the i*M*s and i*SD*s did not vary by gender (*p*s > 0.05), they did covary with age, as older adolescents had higher scores (i*M*: *r* = .27, *p* = .01) and fewer fluctuations (i*SD*: *r *= -.19, *p* = .04).

Multilevel models (nesting siblings within families) were then used to examine how i*M*s and i*SD*s were associated with impulsive behavior, accounting for gender and age. Higher i*M*s were associated with less positive urgency, *b *= -0.02(0.01), *p* = .03, and less negative urgency, *b *= -0.02(0.01), *p* = .04, suggesting that adolescents who had higher average daily IC tended to make less impulsive choices during extreme emotions. Higher i*SD*s were associated with lack of perseverance, *b* = 0.03(0.01), *p* = .03, even after controlling for i*M*s; this suggests that adolescents who had more variable IC were more likely to give up during difficult tasks. No significant links were found between daily IC and lack of premeditation or sensation seeking. Analyses were repeated excluding the first week of skewed assessments, and inferences were the same; see the supplemental materials.

## Illustrative Adolescent-Specific Network Analyses

**Fig. 5:**
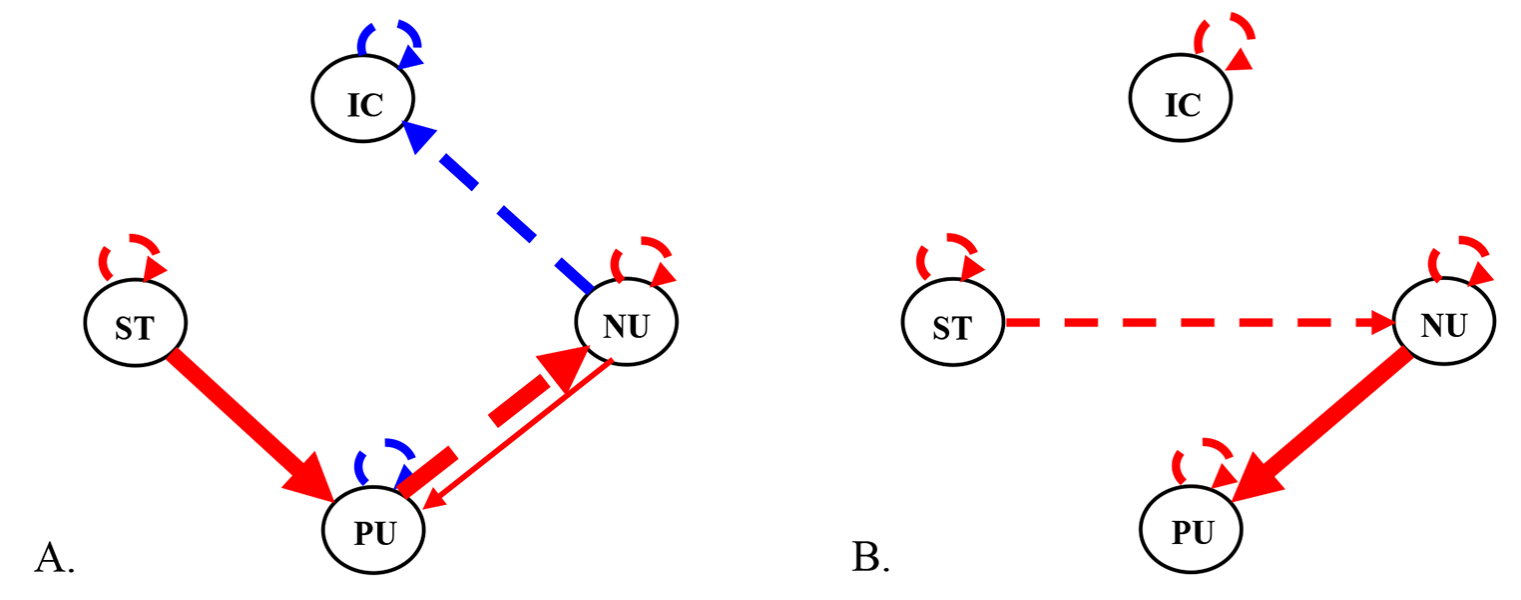
Illustrative person-specific GIMME networks of 100-day inhibitory control (IC), negative urgency (NU), positive urgency (PU), and social time (ST) for two adolescents matched on gender, age, and pubertal status. Circles represent variables. Solid lines represent contemporaneous (i.e., same-day) relations between variables, and dashed lines represent first order lagged (i.e., next-day) relations between variables. Blue lines represent inverse relations, and red lines represent positive relations. Line thickness corresponds to the magnitude (i.e., person-specific beta weight) of the relation. (**A**) Illustrative person-specific network for the adolescent (male, 11.42 years old, PDS = 2.00) who used substances during the 100-day study, *χ*^*2*^(14) = 16.68, *p* = .27, CFI = 0.96, NNFI = 0.93, RMSEA = 0.04, SRMR = 0.08. Beside the autoregressive effect, IC centrality is 1. (**B**) Matched person-specific network for the adolescent (male, 11.83 years old, PDS = 2.40) who did not use substances during the study, *χ*^*2*^(18) = 21.50, *p* = .26, CFI = 0.98, NNFI = 0.97, RMSEA = 0.04, SRMR = 0.05. Beside the autoregressive effect, IC centrality is 0. GIMME: Group Iterative Multiple Model Estimation; PDS: Pubertal Development Scale; CFI: Comparative Fit Index; NNFI: Non-Normed Fit Index; RMSEA: Root Mean Squared Error of Approximation; SRMR: Standardized Root Mean Squared Residual

All adolescent-specific GIMME models fit the data well according to average indices: CFI = 0.99, NNFI = 1.00, RMSEA = 0.02, SRMR = 0.06. Surprisingly, there were no group- or subgroup-level relations (besides the specified autoregressions), indicating substantial heterogeneity among adolescents who used and did not use substances during the 100 days. Figure 5A shows the network of an adolescent (11.42-year-old male) who used alcohol. Circles represent variables and directed arrows represent relations between those variables; solid lines reflect same-day relations, dashed lines reflect next-day relations, red lines reflect positive relations, and blue lines reflect negative relations. Thus, this adolescent’s network contains eight relations, and only four were not specified *a priori* (i.e., the autoregressives). There is a positive, same-day relation between social time and positive urgency, indicating more impulsive behaviors due to positive emotions on days he was more social. There is also a pair of relations between positive and negative urgency, suggesting that negative urgency is related to same-day increases in positive urgency that propagate across time. Finally, there is an inverse, lagged relation between negative urgency and IC suggesting that increases in negative urgency are associated with IC declines the next day. This could reflect a downstream cognitive consequence of actions taken to alleviate unwanted emotions. Figure 5B shows the network of the matched adolescent (11.83-year-old male) who did not use substances. His network is less dense, with only two relations that are not autoregressives. Like the adolescent in Fig. 5A, there is a positive, same-day relation between negative and positive urgency. There is an additional positive, lagged relation between social time and negative urgency, though, potentially suggesting that his social interactions are related to next-day increases in negative urgency. Interestingly, however, his daily IC was not linked to externalizing or social experiences.

It is important to emphasize that the adolescents in Fig. 5 do not characterize the all the adolescents in the sample nor do they characterize all the adolescents who used substances or those who did not in the sample; otherwise, the relations would have appeared at the group- or subgroup-level. Nonetheless, follow-up analyses suggested that adolescents who reported using substances tended to have more relations involving IC (i.e., greater IC centrality) compared to adolescents who did not use substances, *t*(22) = -2.24, *p* = .05, *d* = 0.74. Most (60%) relations with IC were negative (i.e., worse IC was associated with more negative urgency), suggesting that IC was more salient for the daily experiences of substance users.

## Discussion

IC is often conceptualized as a relatively stable construct, but growing evidence suggests that cognition varies across contexts and time (Brose et al., 2014; Sliwinski et al., 2006). Although this has significant implications for adolescent externalizing behaviors, the relevant literature has been stagnated by a lack of suitable intensive longitudinal cognitive assessments. Thus, the present study considered the extent to which adolescent IC – captured by 100 novel daily measurements – fluctuates from day-to-day in ways that are meaningfully associated with impulsivity and differ by the daily experiences of adolescent who do and do not use substances.

### IC Can Be Measured Daily

Findings suggest that the novel IC task is a reliable and valid method for assessing daily cognitive processes related to inhibition. Parallel forms reliability was determined by comparing *M*s and *SD*s across days and calculating ICCs. The 100-day distribution of scores was non-normal: Specifically, IC performance increased over the first week before stabilizing, which may be due to initial task-related learning. Indeed, when the first seven days were excluded, IC scores from the remaining 93 days approximated a normal distribution. Moreover, variation across participants increased over the course of the study until about day 70, indicating increasing then plateauing between-person differences in IC. This is consistent with the notion that contextual demands can reduce individual differences (Miller et al., 2006), with the novelty of the study being an initially constraining context. ICCs also indicated that individuals’ scores displayed moderate reliability across days (Bartko, 1966). This is aligned with expectations for daily studies of cognition - prior work has suggested moderate ICCs are likely well-suited to intensive longitudinal research, as they reflect both between-person stability of assessments and potentially true variation in those assessments within a person over time (Bolger & Laurenceau, 2013).

Notably, most other daily cognition validation studies have not shown similar first-week effects, although there was evidence for learning in a mental rotation task across a 75-day study (Kelly & Beltz, 2021), and there was a short-term practice effect for executive function over the first 4 days of a recent 2-week study (Wang et al., 2021). Thus, the current findings may reflect something unique about executive function (or repeated assessments of it), as IC is typically thought to be a subdomain. Findings could also reflect something unique about adolescence. Younger participants may need more time to become accustomed to intensive assessments (in which case, the first week could reflect noise), or adolescents may be more flexible and malleable thinkers compared to young and older adults (Laube et al., 2020), who have been the participants in most intensive longitudinal studies (in which case, the first week could reflect learning). This is an exciting area for future research.

Clearly, this finding has significant implications for the design and implementation of future intensive longitudinal research as well. Quite a few ‘intensive’ studies are 14 days long (McNeish et al., 2021), which means that their first half could reflect task-related noise or learning effects rather than reflections of daily experiences. Extended training opportunities may be necessary in future studies, and data analytics that explicitly consider stationarity should be used. For instance, daily data detrending was used in this paper’s network analyses and other studies have excluded the first day (or several days) of data collection (e.g., Kelly & Beltz 2021), but these approaches are debatable, and no consensus exists. If more than 14 days of data are available, then continuous time and other models of non-stationarity could be employed, which is an area ripe for future applications (Zhu et al., 2021).

Validity was assessed via correlations of daily IC with standard baseline measures (across participants, separately for each day). As expected, correlations with cognitive measures (i.e., working memory and attentional control) and age were low to moderate, but were always positive and were replicated in multilevel analyses adjusting for sibling dependencies; this provides some evidence of convergent validity. Also as expected, perceptual sensitivity and gender were not associated with IC, providing some evidence of divergent validity.

As higher correlations between daily IC and baseline cognition might have been expected, the reported moderate correlations could reflect some degree of unreliability or some influence of daily experiences. IC and working memory are distinct executive functioning skills that continue to differentiate during adolescence (Miyake & Friedman, 2012), and low correlations (*r* = 0.20 - 0.30) are common in nomothetic and population-level studies (Chaku et al., 2022; Ferguson et al., 2021). Attentional control was also measured via self-report and low correlations are expected with cross-modality measures (Snyder et al., 2021). In task-based measures, participants provide ‘objective’ data with narrowly defined goals, but in self-report measures, participants provide ‘subjective’ data about how they broadly utilize cognitive skills across situations and time (Dang et al., 2020). One exciting direction for future intensive longitudinal research involves assessing multiple inhibitory control tasks, or even other executive functioning tasks. Indeed, a recent study assessing multiple daily executive function tasks over two weeks found that about 40% of daily variation in the latent construct could be attributed to within-person variability (Wang et al., 2021). Thus, future work could consider additional psychometric properties of daily IC or even *individual differences in the structure of executive function* via multilevel or person-specific factor analyses, respectively (Nesselroade & Ford, 1985; Vogelsmeier et al., 2019).

Alternatively, moderate correlations between daily and baseline measures could reflect true variation. Indeed, unreliability and true daily fluctuation would both appear as occasion-to-occasion variation in daily data. The IC task was developed specifically to capture adolescents’ *daily* cognition. It was expected to fluctuate and change potentially in concert with adolescent experiences (Molenaar, 2004; Nesselroade, 1991), whereas existing, standard cognitive measures assume stability across contexts and that variation over short periods of time is merely noise (Bielak et al., 2017). Given these conceptual differences, it is not surprising that daily IC exhibited low-to-moderate correlations with baseline cognition. They shared some degree of common variance, but daily IC ultimately provided distinct, complementary information. As this measure is openly available (https://osf.io/9yabr/), there is ample opportunity for future work to examine the extent to which IC fluctuations are *truly* explained by daily experiences.

### Daily Fluctuations in IC Matter for Impulsive Behaviors

IC exhibited significant fluctuations across days. Gender was not associated with fluctuations, but older adolescents demonstrated fewer fluctuations than younger adolescents. This aligns with an extant literature showing that intraindividual variability decreases from childhood to adolescence, as cognitive skills become more efficient and stable (Dykiert et al., 2012; Hultsch et al., 2011). Thus, intraindividual variability may contain valuable information about development (Lövden et al., 2013; Tamnes et al., 2012) and IC fluctuations (i.e., i*SD*) may be a developmental marker – a construct independent of an individual’s level of IC (i.e., i*M*).

Links between daily IC and baseline impulsivity support this notion. Notably, fluctuations in IC were uniquely associated with greater lack of perseverance, suggesting that adolescents whose IC levels varied more across days were also more likely to give up during difficult tasks. This was true even when controlling for average IC, indicating that fluctuations in IC provide distinct information about constructs related to impulsivity. Adolescents who experience more fluctuations in IC may live in more chaotic environments or may be more susceptible to environmental changes (e.g., less sleep, more stress); thus, variability may be a developmental marker of stress reactivity or vulnerability (Nesselroade, 1991). Understanding how these fluctuations are linked to everyday experiences is just beginning to be realized and future time-indexed, multivariate studies would be well-poised to investigate these questions. Beyond fluctuations and perseverance, higher average IC was also associated with less positive and negative urgency, suggesting that average IC – assessed in the context of adolescents’ everyday lives – may be particularly relevant for impulsive behaviors that occur in emotional or motivational situations. This nomothetic inference is consistent with the larger literature suggesting that IC is particularly salient for externalizing behaviors (Heffer & Willoughby, 2021).

### Adolescents Are Unique

Research has largely focused on characterizing the ‘average’ adolescent, but averages cannot characterize the biopsychosocial experiences that are unique to a single adolescent (Molenaar, 2004), so a person-specific approach (i.e., GIMME) was used to accurately model developmental heterogeneity among the daily data of adolescents who used substances and those who did not (Gates & Molenaar, 2012; Henry et al., 2019). Even though GIMME prioritized commonalities across all adolescents and within subgroups during model building, none were found; in fact, no two individuals shared the exact same pattern of associations. This highlights the significant heterogeneity among adolescents who use substances and how links between IC and externalizing behaviors may unfold in unique ways for unique youth. As with all approaches, though, some caution is warranted because several features of the dataset (e.g., power or measurement intervals) could impact estimation of individual-level network features (see Weigard et al., 2021).

Despite this, indices that quantified the importance of each variable in the adolescent-specific networks (i.e., centrality) suggested that IC played a more important role in the networks of adolescents who used substances than those who did not. Thus, IC may represent a hub that integrates and distributes information within and between externalizing and social behaviors for adolescents who use substances. Indeed, nomothetic studies have found that IC explains more variance (i.e., plays a central role) in externalizing behaviors than working memory or other cognitive skills (Young et al., 2009). Future intensive longitudinal work could explore the specific influence of IC on externalizing behaviors, for example, by leveraging the direction of relations with IC to determine whether it is more likely to influence (i.e., out-degree) or be influenced by (i.e., in-degree) other network behaviors. Such directional inferences require specialized network analyses, such as the multiple solutions version of GIMME (Beltz & Molenaar, 2016).

Although this GIMME analysis only focused on a subset of adolescents who used substances in order to emphasize insights into externalizing behaviors, it nonetheless illustrates one way in which intensive longitudinal data can be used to inform future personalized studies of externalizing behaviors. Clinical science already has a rich history of examining externalizing behaviors in context via EMA (reviewed in Votaw & Witkiewitz 2021), and future research could benefit from intensively measuring individuals during interventions, paving the way forward for individualized, tailored, and even adaptive treatments (Ginexi et al., 2014). For example, a randomized clinical trial used 2-week pre-treatment EMA data to develop individualized cognitive behavioral therapy plans for individuals struggling with substance use, and those strategies were utilized during temptation, according to post-treatment EMA data (Litt et al., 2009). As EMA typically has fewer and shorter measures than the 100-day intensive longitudinal study described here, it is easy to envision the extensive opportunities for personalization in both basic and applied science.

There are also new and exciting questions that could be answered with personalized analyses of intensive longitudinal data. For instance, although relations between cognition and other behaviors (e.g., sleep, affect, and mood) are well-established at the between-person level, there is limited and somewhat contradictory, evidence at the within-person level (Hawks et al., 2022; Neubauer et al., 2019). As reliable and valid intensive cognitive assessments (like the IC task used here) become increasingly available, though, within-person insights may become clear. Another important direction concerns how intraindividual variation in IC (e.g., its stability or behavioral network centrality) over development is related to interindividual differences in sensation seeking and other externalizing behaviors (e.g., substance use, antisocial behavior, or conduct disorders) known to normatively shift across adolescence. IC’s daily fluctuation or centrality in a network may be a marker of development or maturation, which could be revealed in measurement burst designs that incorporate intensive measurement (e.g., over days or weeks) with traditional longitudinal measurement (e.g., annually; Sliwinski 2008).

### Study Considerations

This study had several limitations that should be considered. First, data collection was completed during the novel coronavirus-19 (COVID-19) pandemic; inferences may have been impacted by this period of heightened instability. All testing was conducted during the pandemic, though, so effects were similarly distributed across adolescents, and intensive longitudinal studies emphasize intraindividual *variation* (not necessarily *levels*).

Second, because all testing was conducted virtually, a small subset of days (representing < 0.5 of all data) may have been affected by technical difficulties (e.g., screen freezing or spotty connectivity). Further, it was difficult to examine potential impacts of the timing of daily survey completion (e.g., whether adolescents completed a daily survey the next morning) due to software limitations. This is somewhat common in intensive longitudinal studies (Keijsers et al., 2022). Future research could use local application-based assessments (that do not require Internet connectivity) or ask participants to report technical difficulties, but these alternatives may be costly or burdensome for participants.

Third, the sample (*N* = 106) was smaller than in other validation studies (Collins et al., 2016), including some that included intensive longitudinal cognitive measures (e.g., Kelly & Beltz 2021), but the 100-day time series was longer than other intensive longitudinal studies, and retention was excellent. Of recruited adolescents, 61% completed over 80% of the daily diaries (with an average response rate of 94%). This completion rate is like other intensive longitudinal studies using adult samples (Teague et al., 2018; Wright et al., 2019) and better than others using adolescent samples (Keijsers et al., 2022).

Fourth, although the sample was generally reflective of the surrounding area, there was limited race/ethnic diversity, with most adolescents endorsing White race/ethnicities (75.5%); thus, findings may not be generalizable to all adolescents and must be replicated in larger and more representative samples of uniquely diverse youth. Further, although excluded participants were similar in age, gender, and race/ethnicity to included participants, they did report more impulsive behaviors and lower cognitive skills, suggesting that study findings may also have limited generalizability to participants with high externalizing problems. This is unfortunately a common finding in traditional cross-sectional and longitudinal research even though other intensive longitudinal studies do not systematically report associations between psychological characteristics and participant retention (see Ewing et al., 2018). Although not without trade-offs, ways to facilitate retention include using shorter daily surveys, collecting data for fewer days, implementing planned missingness or related designs (Yuan et al., 2020), and leveraging passive sensing data (Ponnada et al., 2022). The present study was designed to be 100 days because it provided enough within-person power to estimate person-specific models with 20% missing data (Rankin & Marsh, 1985); with a 30-day study, fewer data would likely be missing, but person-specific analyses could not be conducted.

Fifth, identical measures were not used at baseline and in the daily assessments, limiting direct comparisons, but this is true in almost all intensive longitudinal studies, as measures must be adapted for repeated use (Chaku & Beltz, 2022). Finally, only adolescents (e.g., not parents) reported on daily externalizing behaviors; there was low endorsement of externalizing behaviors generally, and clinically-significant externalizing disorders were not directly assessed. Only 12 adolescents endorsed using any type of substance during the study and rates of impulsive behaviors were generally low. This could be related to adolescent ability to access substances during the COVID-19 pandemic. Nonetheless, these are important avenues to explore in future research. Parent reports would be especially relevant for younger samples and replicating these analyses in clinical samples could shed light on patterns unique to those on the externalizing spectrum, as daily IC may represent different constructs in those with, for example, substance use disorders versus antisocial behavior.

## Conclusions

Externalizing behaviors normatively increase during adolescence in ways that accentuate individual differences and implicate IC (Young et al., 2009). Yet, IC – and its expression across different contexts and times – is thought to be stable even though growing evidence suggests it may vary within individuals (Brose et al., 2014; Sliwinski et al., 2006). Thus, single assessments that describe ‘average’ adolescents may not accurately capture the lived experiences of individuals, including their daily highs and lows. In an attempt to fill this knowledge gap, this intensive longitudinal study followed over 100 adolescents for 100 days while they completed a novel, openly available, IC task. The task was generally found to be reliable and valid, with daily levels of IC and fluctuations in IC associated with individual differences in impulsive behaviors. Moreover, an adolescent-specific network analysis comparing adolescents who used substances over the 100 days to those who did not revealed that IC was central to the daily interplay among impulsivity and adolescent social experiences, but only for substance users. Thus, the new IC measure – and the illustration of its use in adolescent intensive longitudinal research – encourages future innovation in the investigation of adolescent-specific externalizing behaviors.

### Electronic Supplementary Material

Below is the link to the electronic supplementary material.

Supplementary Material 1

## Footnotes

1 This included adolescents with transgender, gender non-conforming, and genderfluid identities.

2 Adolescents who were at least 18 years old could opt to receive their emails directly.

3 Adolescents were allowed to unclick a response and select a new one.

4 Adolescents who reported non-cisgender identities (*n* = 4) were excluded from these analyses.
