# Supplementary material for: 100 days of Adolescence: Elucidating Externalizing Behaviors Through the Daily Assessment of Inhibitory Control
Source: Res Child Adolesc Psychopathol. 2023 Jul 5;52(1):93–110. doi: 10.1007/s10802-023-01071-y (PMC10787911; doi:10.1007/s10802-023-01071-y)
Supplement: Supplementary file 1 — Supplementary Material 1 [file 10802_2023_1071_MOESM1_ESM.docx]

**Supplemental Materials for “100 days of adolescence: Elucidating externalizing behaviors through the daily assessment of inhibitory control”**

[AUTHORS NAME REDACTED]

**Methods**

**Images of the Daily Inhibitory Control Task**


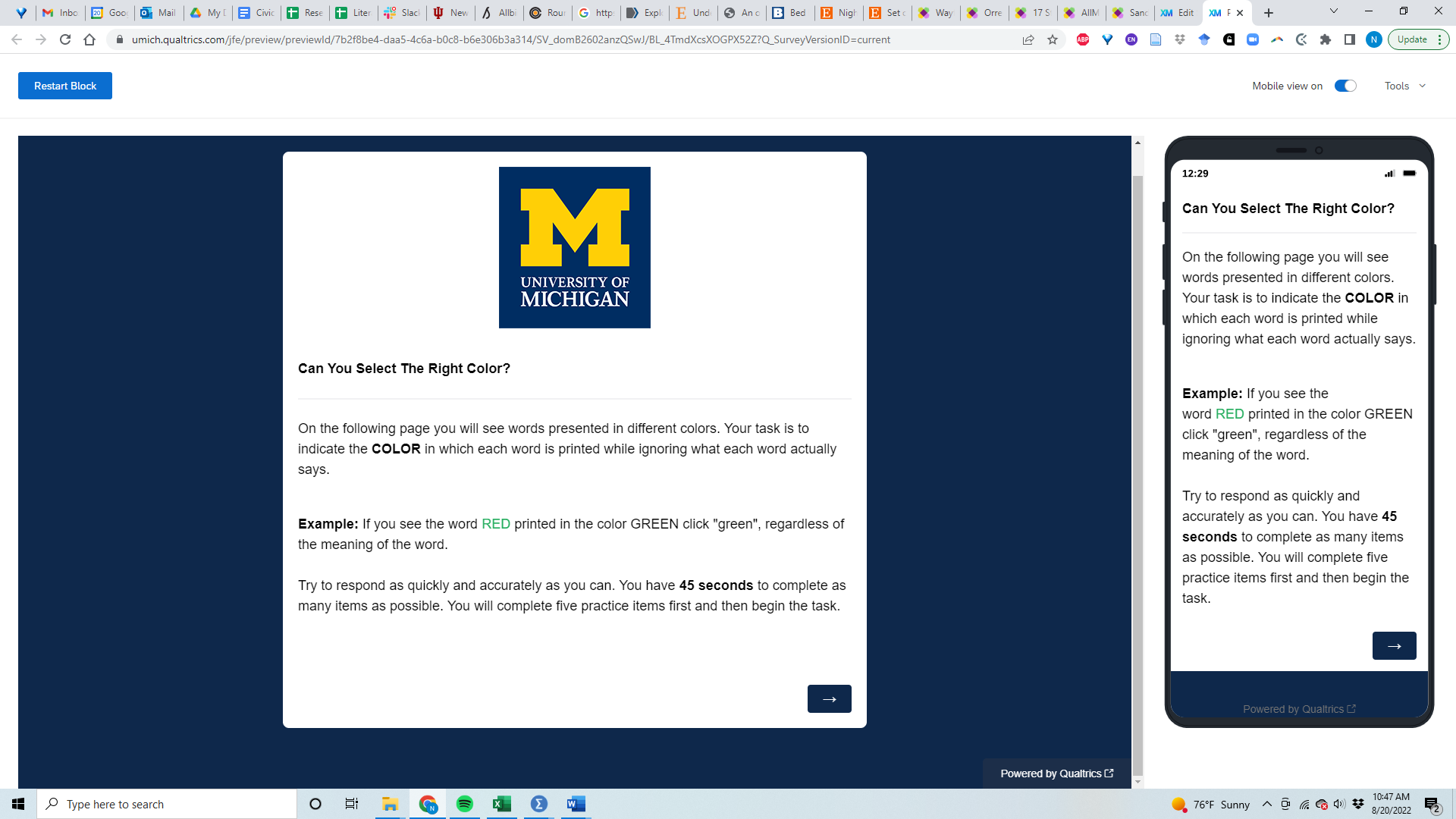


*Figure S1*. IC task instructions in Qualtrics. The computer version of the instructions is presented on the left, and the mobile version of the instructions is presented on the right.


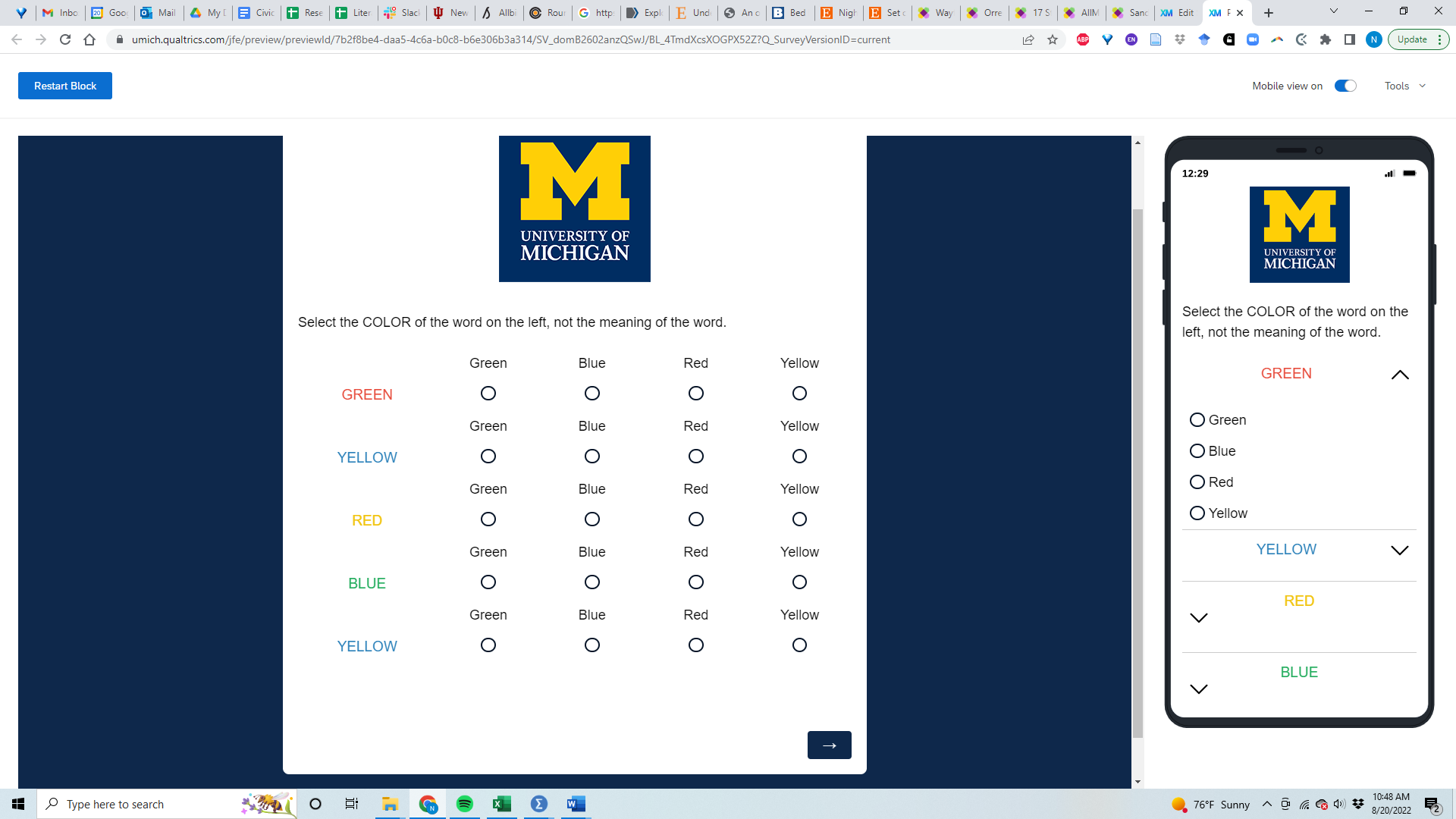


*Figure S2*. IC training block in Qualtrics. Participants were instructed to select the answer choice that corresponded to the color of the word on the left and not the meaning of the word by clicking on the correct radio button on the right. The computer version of the training block is presented on the left, and the mobile version of the training block is presented on the right.


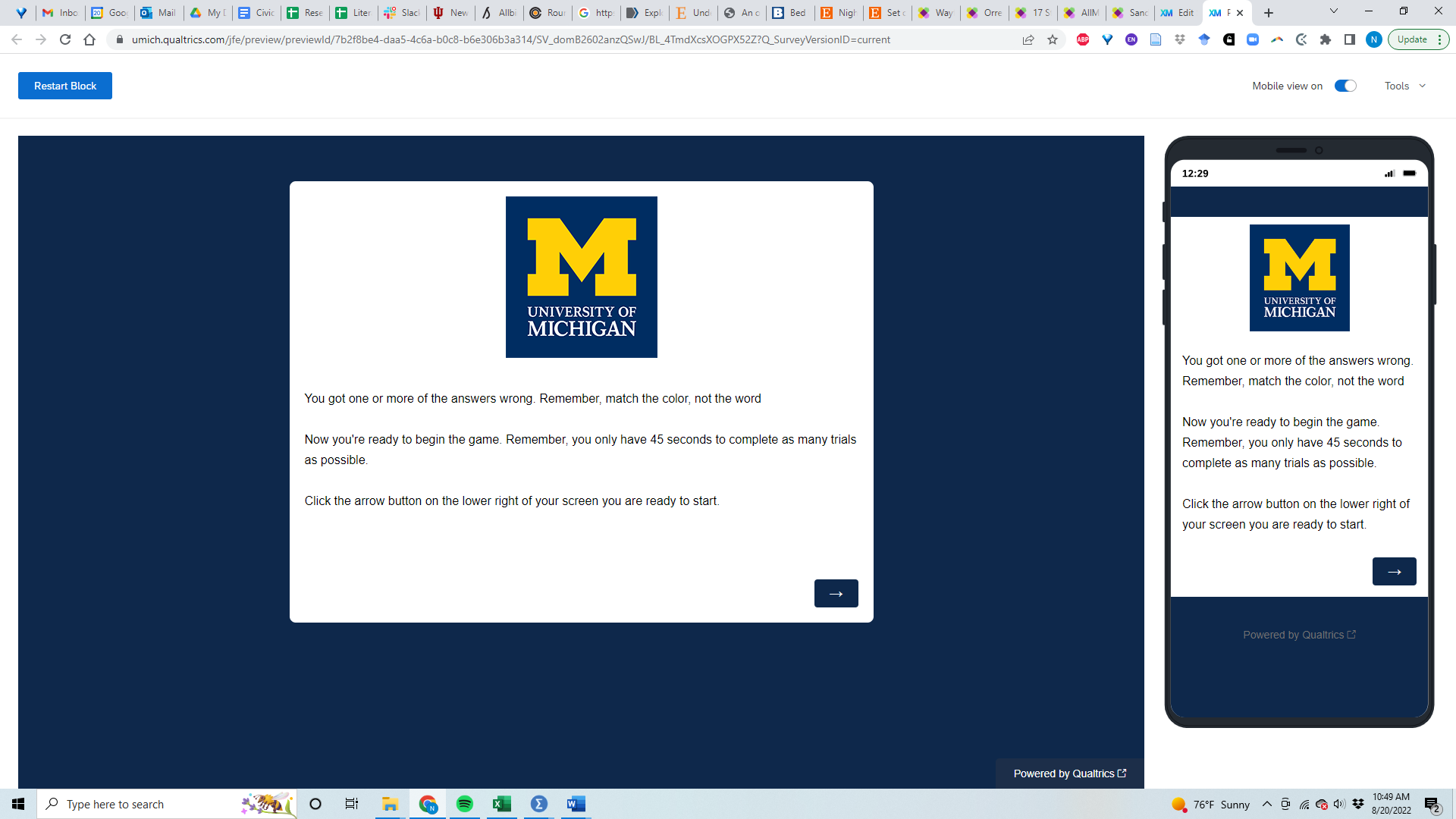


*Figure S3*. IC training feedback in Qualtrics. Feedback was given to participants and task instructions were repeated for all participants (regardless of performance). The computer version of the training feedback is presented on the left, and the mobile version of the training feedback is presented on the right.


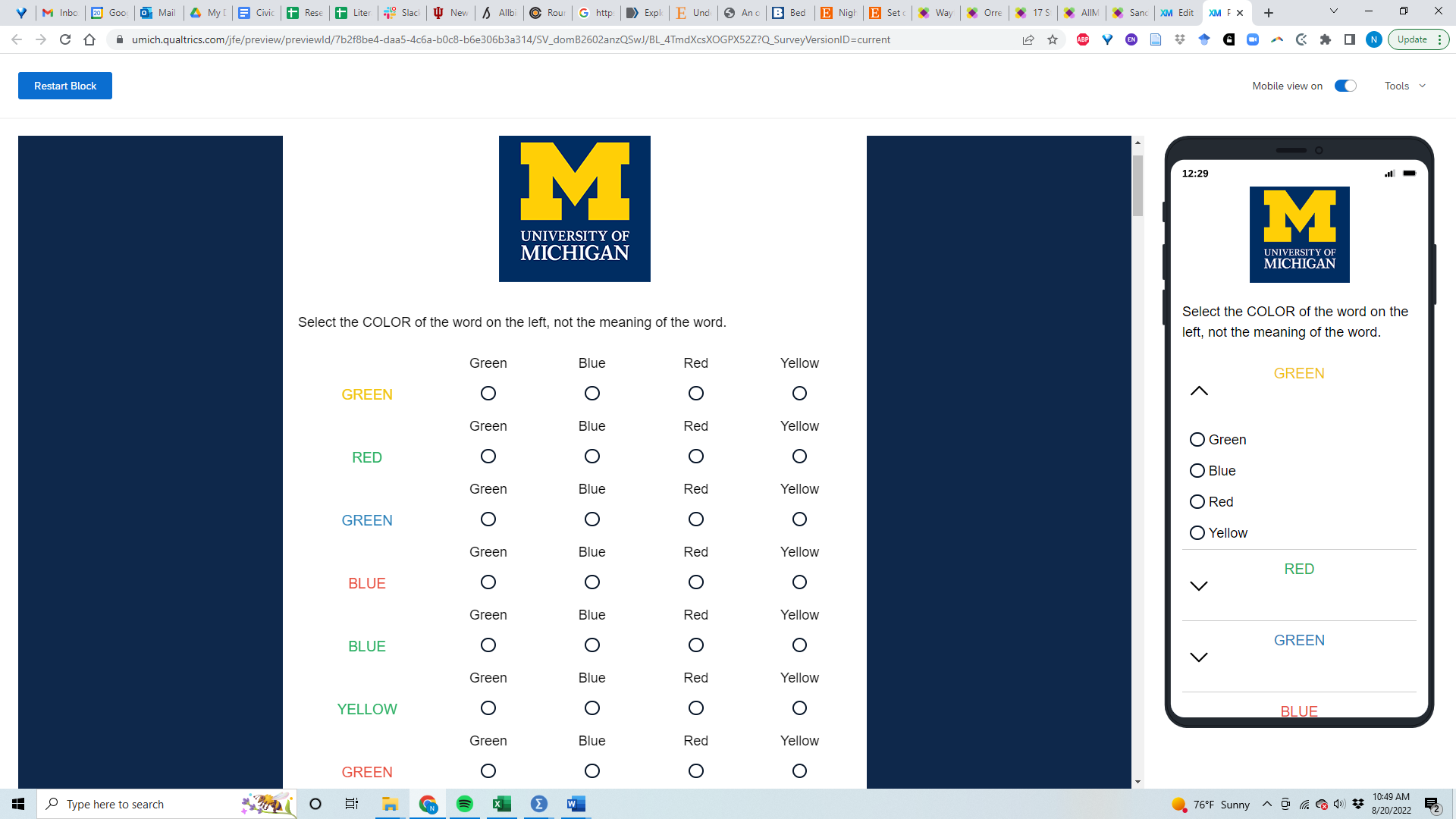


*Figure S4*. IC task in Qualtrics for day 1. Participants had 45 seconds to select answer choices that corresponded to the color of the word on the left and not the meaning of the word by clicking on the correct radio button on the right. Participants could complete the trials in any order, and they could scroll down to answer additional trials (up to 100). The screen automatically advanced after 45 seconds. The computer version of the task is presented on the left, and the mobile version of the task is presented on the right.

**Daily measures**

The illustrative adolescent-specific network analyses were conducted using group iterative multiple model estimation (GIMME; Gates & Molenaar, 2012), and they included four variables, assessed daily for 100 days: IC, externalizing behaviors (i.e., positive urgency and negative urgency), and social experiences. The IC task was reviewed in the main manuscript, but the other daily variables were only reviewed briefly, so they are elaborated on here.

Daily externalizing behaviors were assessed using the Short UPPS-P Impulsive Behavior Scale (SUPPS-P; Cyders et al., 2014). The SUPPS-P is like the standard UPPS-PC (Whiteside & Lynam, 2001) and contains the same subscales. It is only 20 items long, though, making it ideal for intensive measurement (e.g., Lydon-Staley et al., 2020). The positive urgency subscale and the negative urgency subscale (were used in this study’s network analyses because they reflect a continuum of behaviors likely to occur in the everyday life of an adolescent.

Social experiences were assessed using an adapted version of the social activities reported in a daily time use questionnaire (that was originally conducted over-the-phone; McHale et al., 1999). In this study, adolescents indicated how much time they spent doing various activities (e.g., helping around the house, completing homework, or doing hobbies). The amount of time spent visiting or hanging out was used as the indicator of daily social time in this paper. Specifically, adolescents were asked everyday whether they visited or hung out (with others at their house or at a friend or relative’s house). If they reported that they did, then they also indicated (via a continuous slider) how much time they spent visiting or hanging out (from 0 to 100 minutes). If they spent more than 100 minutes hanging out, then adolescents were instructed to move the slider to 100 minutes.

**Identification of substance users**

As part of the intensive longitudinal study, youth were asked to report on their substance use every day for 100 days. Specifically, adolescents were asked about their caffeine, cigarette/tobacco, marijuana, and alcohol intake that day (e.g., “How many marijuana cigarettes or smokeless marijuana servings did you have today?”). Adolescents responded by typing in their number of servings for that day. Then, they reported how typical that number of servings was for them (e.g., “How does this compare to the amount you typically use on this day of the week?”) on a 5-point scale (1=*Much more than I typically use* to 5=*Much less than I typically use)*. Twelve adolescents reported using substances other than caffeine (i.e., cigarette/tobacco, marijuana, or alcohol) at least once over the course of the 100-day study; they composed the substance use subgroup. Descriptive statistics for this subsample are reported in Table S1.

**Matching criteria**

Each of the 12 adolescents in the substance use subgroup were matched on gender, age, and pubertal status to an adolescent who did not report using any substances during the 100-day study. Case-control matching was conducted in SPSS (version 28) which employs fuzzy matching based on the tolerance specified for each match parameter (Kim & Baek, 2016). Tolerance was set to zero for gender and 1 for age and pubertal status (i.e., to be matched with a substance user, an adolescent had to be the same gender, within 1 year of their age, and within 1-point of their PDS score). Matches were made without replacement and prioritized exact matches.

Gender was assessed with a single question (i.e., “What is your gender?”). Answer choices included *Boy*, *Girl*, *Transgender, Gender Nonconforming,* and *Preferred response not listed*. If adolescents selected *Preferred response not listed,* they were asked to specify their gender identity in a text box. Age was derived by subtracting the start-date of the study from adolescent birthdates, which were provided by their parent or legal guardian. Pubertal status was assessed using the Pubertal Development Scale (PDS; Petersen et al., 1988). The PDS is composed of three general items concerning adolescent changes in height, body hair, and skin and of two gender-specific items concerning facial hair growth and voice changes in boys or breast development and menarche in girls. For almost all questions, youth rated their physical development on a four-point scale (1=*Has not yet begun* to 4=*Is complete*). Menarche, however, was rated on a two-point scale (1=*No* or 4=*Yes*). Pubertal status was the average of all PDS items and was calculated separately for boys and girls. Thus, adolescents with scores closer to one were prepubertal or just beginning puberty, whereas adolescents with scores closer to 4 were near the end of puberty or post-pubertal. Adolescents received the version of the PDS that corresponded to their sex, as reported by their parent or legal guardian, but all adolescents were reminded that they could skip items they felt did not apply to them. Descriptive statistics for this subsample are reported in Table S1. As can be seen, both subsamples (i.e., substance users and non-users) had equal numbers of boys and girls and were highly similar in age and mid-pubertal status.

**Table S1.** Descriptive statistics for substance users and matched non-substance users

|  | Substance users (*n* = 12) | | Non-substance users (*n* = 12) | |
| --- | --- | --- | --- | --- |
|  | *n* | *%* | *n* | *%* |
| Gender ^a^ |  |  |  |  |
| Male | 6 | 50% | 6 | 50% |
| Female | 6 | 50% | 6 | 50% |
|  | *M* | *SD* | *M* | *SD* |
| Age | 13.78 | 2.08 | 13.90 | 2.11 |
| PDS | 2.73 | 0.84 | 2.41 | 0.85 |

Notes. M = Mean; SD = Standard deviation. ^a^Non-cisgender identities were not endorsed by adolescents in these subsamples*.*

**Results**

**Correlations of Daily IC with Baseline Cognitive Measures**

Table S2 shows the correlations (*r*) of daily IC with working memory and attentional control, presented separately for each day. Table S3 shows the same analyses assessed via multilevel models (nesting adolescents within families). Specifically, working memory was regressed onto each day’s IC (100 models total), and in separate models, attentional control was also regressed onto each day’s IC (100 models total). Daily IC was the fixed effect and family membership was a random intercept in the multilevel models. Notice that the pattern of significance between the statistics in Tables S2 and S3 are similar, suggesting that family dependencies did not strongly influence the psychometric findings.

**Table S2.** Correlations between daily IC and standard cognitive measures

| Day | IC &  Working Memory  Correlation *(R)* | IC &  Attentional Control Correlation *(R)* |  | Day | IC &  Working Memory  Correlation *(R)* | IC &  Attentional Control Correlation *(R)* |
| --- | --- | --- | --- | --- | --- | --- |
| Day 1 | 0.06 | 0.13 |  | Day 51 | 0.17* | 0.25 |
| Day 2 | 0.21* | 0.27 |  | Day 52 | 0.21* | 0.26 |
| Day 3 | 0.06 | 0.12 |  | Day 53 | 0.13 | 0.37 |
| Day 4 | 0.13 | 0.21 |  | Day 54 | 0.09 | 0.28 |
| Day 5 | 0.23* | 0.23 |  | Day 55 | 0.21* | 0.35 |
| Day 6 | 0.08 | 0.17 |  | Day 56 | 0.23* | 0.09 |
| Day 7 | 0.19* | 0.27 |  | Day 57 | 0.15 | 0.24 |
| Day 8 | 0.26* | 0.25 |  | Day 58 | 0.31** | 0.24 |
| Day 9 | 0.17* | 0.14 |  | Day 59 | 0.15 | 0.22 |
| Day 10 | 0.28** | 0.23 |  | Day 60 | 0.25** | 0.22 |
| Day 11 | 0.13 | 0.26 |  | Day 61 | 0.01 | 0.16 |
| Day 12 | 0.28** | 0.28 |  | Day 62 | 0.33*** | 0.17 |
| Day 13 | 0.30*** | 0.20 |  | Day 63 | 0.04 | 0.19 |
| Day 14 | 0.25* | 0.12 |  | Day 64 | 0.09 | 0.12 |
| Day 15 | 0.23* | 0.30 |  | Day 65 | 0.37*** | 0.11 |
| Day 16 | 0.25** | 0.20 |  | Day 66 | 0.04 | 0.17 |
| Day 17 | 0.24 | 0.20 |  | Day 67 | 0.09 | 0.14 |
| Day 18 | 0.08 | 0.21 |  | Day 68 | 0.23* | 0.12 |
| Day 19 | 0.15 | 0.27 |  | Day 69 | 0.36*** | 0.13 |
| Day 20 | 0.11 | 0.34 |  | Day 70 | 0.21* | 0.11 |
| Day 21 | 0.03 | 0.24 |  | Day 71 | 0.29** | 0.15 |
| Day 22 | 0.06 | 0.24 |  | Day 72 | 0.10 | 0.15 |
| Day 23 | 0.11 | 0.20 |  | Day 73 | 0.13 | 0.22 |
| Day 24 | 0.09 | 0.27 |  | Day 74 | 0.15 | 0.19 |
| Day 25 | 0.01 | 0.12 |  | Day 75 | 0.36*** | 0.25 |
| Day 26 | 0.03 | 0.27 |  | Day 76 | 0.29** | 0.11 |
| Day 27 | 0.02 | 0.12 |  | Day 77 | 0.27** | 0.13 |
| Day 28 | 0.07 | 0.21 |  | Day 78 | 0.26** | 0.21 |
| Day 29 | 0.05 | 0.25 |  | Day 79 | 0.14 | 0.15 |
| Day 30 | 0.11 | 0.21 |  | Day 80 | 0.02 | 0.29 |
| Day 31 | 0.07 | 0.16 |  | Day 81 | 0.18* | 0.25 |
| Day 32 | 0.11 | 0.36 |  | Day 82 | 0.12 | 0.12 |
| Day 33 | 0.26* | 0.18 |  | Day 83 | 0.26* | 0.24 |
| Day 34 | 0.19* | 0.23 |  | Day 84 | 0.03 | 0.19 |
| Day 35 | 0.06 | 0.22 |  | Day 85 | 0.19* | 0.13 |
| Day 36 | 0.04 | 0.35 |  | Day 86 | 0.21* | 0.19 |
| Day 37 | 0.09 | 0.21 |  | Day 87 | 0.18* | 0.18 |
| Day 38 | 0.06 | 0.26 |  | Day 88 | 0.33*** | 0.12 |
| Day 39 | 0.19* | 0.27 |  | Day 89 | 0.09 | 0.11 |
| Day 40 | 0.22* | 0.23 |  | Day 90 | 0.12 | 0.10 |
| Day 41 | 0.19* | 0.29 |  | Day 91 | 0.06 | 0.12 |
| Day 42 | 0.08 | 0.31 |  | Day 92 | 0.29** | 0.12 |
| Day 43 | 0.26** | 0.25 |  | Day 93 | 0.23* | 0.20 |
| Day 44 | 0.02 | 0.16 |  | Day 94 | 0.12 | 0.12 |
| Day 45 | 0.07 | 0.10 |  | Day 95 | 0.30** | 0.01 |
| Day 46 | 0.12 | 0.21 |  | Day 96 | 0.19* | 0.14 |
| Day 47 | 0.03 | 0.23 |  | Day 97 | 0.03 | 0.05 |
| Day 48 | 0.18* | 0.15 |  | Day 98 | 0.26* | 0.09 |
| Day 49 | 0.11 | 0.17 |  | Day 99 | 0.11 | 0.07 |
| Day 50 | 0.15 | 0.11 |  | Day 100 | 0.27** | 0.24 |

**Notes.** IC = inhibitory control. **p<*.05, ***p*<.01, ****p*<.001

**Table S3.** Unstandardized coefficients from multilevel models assessing relations between daily IC (predictor) and standard cognitive measures (outcomes)

| Daily IC | Working Memory | Attentional Control |  | Daily IC | Working Memory | Attentional Control |
| --- | --- | --- | --- | --- | --- | --- |
|  | *B* (*SE*) | *B* (*SE*) |  |  | *B* (*SE*) | *B* (*SE*) |
| Day 1 | .05 (.08) | .01 (.01) |  | Day 51 | .07* (.03) | .01* (.01) |
| Day 2 | .13* (.06) | .06 (.08) |  | Day 52 | .08* (.04) | .01* (.01) |
| Day 3 | .04 (.05) | .01 (.01) |  | Day 53 | .05 (.04) | .02* (.01) |
| Day 4 | .07 (.05) | .01 (.01) |  | Day 54 | .04 (.04) | .01* (.01) |
| Day 5 | .14* (.06) | .02* (.01) |  | Day 55 | .08* (.04) | .02* (.01) |
| Day 6 | .04 (.05) | .01 (.01) |  | Day 56 | .09* (.04) | .004 (.01) |
| Day 7 | .10* (.05) | .02** (.01) |  | Day 57 | .06 (.04) | .01* (.01) |
| Day 8 | .11* (.04) | .02* (.01) |  | Day 58 | .11*** (.03) | .01* (.01) |
| Day 9 | .07* (.03) | .01 (.01) |  | Day 59 | .06 (.04) | .01 (.01) |
| Day 10 | .12** (.04) | .02* (.01) |  | Day 60 | .07** (.03) | .01* (.01) |
| Day 11 | .06 (.05) | .02** (.01) |  | Day 61 | .001 (.04) | .01 (.01) |
| Day 12 | .12** (.04) | .02** (.01) |  | Day 62 | .14*** (.04) | .01 (.01) |
| Day 13 | .10*** (.03) | .01 (.01) |  | Day 63 | .01 (.04) | .01 (.09) |
| Day 14 | .10** (.04) | .01 (.01) |  | Day 64 | .03 (.04) | .01 (.01) |
| Day 15 | .09* (.04) | .02** (.01) |  | Day 65 | .14*** (.03) | .01 (.01) |
| Day 16 | .12** (.04) | .02 (.01) |  | Day 66 | .01 (.04) | .01 (.01) |
| Day 17 | .09* (.04) | .01 (.01) |  | Day 67 | .04 (.03) | .01 (.01) |
| Day 18 | .03 (.04) | .01* (.01) |  | Day 68 | .08* (.03) | .01 (.01) |
| Day 19 | .07 (.05) | .02** (.01) |  | Day 69 | .13*** (.03) | .01 (.01) |
| Day 20 | .06 (.05) | .02*** (.01) |  | Day 70 | .08* (.04) | .01 (.01) |
| Day 21 | .002 (.05) | .02* (.01) |  | Day 71 | .12*** (.03) | .01 (.01) |
| Day 22 | .03 (.04) | .02* (.01) |  | Day 72 | .03 (.04) | .01 (.01) |
| Day 23 | .05 (.02) | .01* (.01) |  | Day 73 | .04 (.04) | .01* (.01) |
| Day 24 | .01 (.04) | .02* (.01) |  | Day 74 | .06 (.04) | .01* (.01) |
| Day 25 | .002 (.04) | .01 (.01) |  | Day 75 | .11*** (.03) | .01* (.01) |
| Day 26 | .05 (.04) | .01* (.01) |  | Day 76 | .10*** (.03) | .004 (.01) |
| Day 27 | .01 (.05) | .01 (.01) |  | Day 77 | .11** (.04) | .01 (.01) |
| Day 28 | .03 (.04) | .01* (.01) |  | Day 78 | .10* (.04) | .01* (.01) |
| Day 29 | .02 (.04) | .02* (.01) |  | Day 79 | .05 (.04) | .01 (.01 |
| Day 30 | .05 (.04) | .01 (.01) |  | Day 80 | .01 (.04) | .02** (.01) |
| Day 31 | .03 (.04) | .01 (.01) |  | Day 81 | .05 (.03) | .01* (.01) |
| Day 32 | .05 (.05) | .02*** (.01) |  | Day 82 | .04 (.04) | .003 (.01) |
| Day 33 | .12* (.05) | .01 (.01) |  | Day 83 | .09* (.04) | .01* (.01) |
| Day 34 | .08* (.03) | .01* (.01) |  | Day 84 | .001 (.03) | .01* (.01) |
| Day 35 | .03 (.04) | .01 (.01) |  | Day 85 | .07* (.04) | .01 (.01) |
| Day 36 | .02 (.05) | .02*** (.01) |  | Day 86 | .06* (.03) | .01 (.01) |
| Day 37 | .03 (.04) | .01 (.01) |  | Day 87 | .06* (.03) | .01 (.01) |
| Day 38 | .02 (.05) | .02* (.01) |  | Day 88 | .12*** (.04) | .01 (.01) |
| Day 39 | .07* (.04) | .02* (.01) |  | Day 89 | .03 (.04) | .003 (.01) |
| Day 40 | .09* (.04) | .01* (.01) |  | Day 90 | .04 (.04) | .003 (.01) |
| Day 41 | .06* (.03) | .02** (.01) |  | Day 91 | .02 (.04) | .01 (.01) |
| Day 42 | .03 (.05) | .02*** (.01) |  | Day 92 | .10** (.03) | .01 (.01) |
| Day 43 | .10** (.04) | .01* (.01) |  | Day 93 | .06* (.03) | .01* (.01) |
| Day 44 | .01 (.04) | .01 (.01 |  | Day 94 | .04 (.03) | .004 (.01) |
| Day 45 | .02 (.04) | .01 (.01 |  | Day 95 | .09*** (.03) | .002 (.01) |
| Day 46 | .05 (.04) | .01 (.01) |  | Day 96 | .08* (.04) | .01 (.01) |
| Day 47 | .01 (.04) | .01 (.01) |  | Day 97 | .01 (.04) | .001 (.01) |
| Day 48 | .06* (.03) | .01 (.01) |  | Day 98 | .10** (.04) | .003 (.01) |
| Day 49 | .04 (.04) | .01 (.01) |  | Day 99 | .04 (.04) | .001 (.001) |
| Day 50 | .06 (.04) | .01 (.01) |  | Day 100 | .09** (.03) | .01 (.01) |

**Notes.** IC = inhibitory control. **p<*.05, ***p*<.01, ****p*<.001

**93-Day Fluctuations in IC**

Due to the skewed IC scores in the first week of the 100-day study, i*M* and i*SD* analyses were repeated for the last 93 days as a sensitivity analysis. Results, however, were generally consistent with those seen in the 100-day assessments reported in the main text. Across adolescents, the i*M* ranged from 12.04 to 56.63, and the i*SD* ranged from 2.69 to 15.93. A one-sample *t*-test indicated that the i*SDs* were significantly different from zero, *t*(105)=29.16, *p*<.001. Similar to the 100-day assessments, i*M* and i*SD*s did not vary by gender (*p*s>.05), but did vary with age. Older adolescents had higher i*M* (*r*=.26, *p*=.01) and fewer fluctuations (i*SD*: *r*=-.20, *p*=.04).

Multilevel models (nesting siblings within families) to assess associations between i*M*s and i*SD*s with impulsive behavior across 93 days were also generally consistent with the findings from the 100-day assessments. Higher i*M*s were associated with less positive urgency, *b*=-.01(.01), *p* =.05, and less negative urgency, *b*=-.02(.01), *p* =.04. Higher i*SD*s were associated with greater sensation seeking, *b*=.03(.01), *p* = .05. Again, no significant links between daily IC and lack of premeditation or perseverance were found.

References

Cyders, M. A., Littlefield, A. K., Coffey, S., & Karyadi, K. A. (2014). Examination of a short English version of the UPPS-P Impulsive Behavior Scale. *Addictive Behaviors, 39*(9), 1372-1376. <https://doi.org/10.1016/j.addbeh.2014.02.013>

Gates, K. M., & Molenaar, P. C. (2012). Group search algorithm recovers effective connectivity maps for individuals in homogeneous and heterogeneous samples. *NeuroImage, 63*(1), 310-319.

Kim, S. Y., & Baek, J. I. (2016). FUZZY matching using propensity score: IBM SPSS 22 Ver. *Journal of the Korean Data and Information Science Society, 27*(1), 91-100.

Lydon-Staley, D. M., Falk, E. B., & Bassett, D. S. (2020). Within-person variability in sensation-seeking during daily life: Positive associations with alcohol use and self-defined risky behaviors. *Psychology of addictive behaviors, 34*(2), 257.

McHale, S. M., Crouter, A. C., & Tucker, C. J. (1999). Family context and gender role socialization in middle childhood: comparing girls to boys and sisters to brothers. *Child Development, 70*(4), 990-1004. <https://doi.org/10.1111/1467-8624.00072>

Petersen, A. C., Crockett, L., Richards, M., & Boxer, A. (1988). A self-report measure of pubertal status: Reliability, validity, and initial norms. *Journal of Youth and Adolescence, 17*(2), 117-133.

Sperry, S. H., Lynam, D. R., Walsh, M. A., Horton, L. E., & Kwapil, T. R. (2016). Examining the multidimensional structure of impulsivity in daily life. *Personality and individual differences, 94*, 153-158.

Tomko, R. L., Solhan, M. B., Carpenter, R. W., Brown, W. C., Jahng, S., Wood, P. K., & Trull, T. J. (2014). Measuring impulsivity in daily life: the momentary impulsivity scale. *Psychological assessment, 26*(2), 339.

Whiteside, S. P., & Lynam, D. R. (2001). The five factor model and impulsivity: Using a structural model of personality to understand impulsivity. *Personality and individual differences, 30*(4), 669-689.
